# Catecholamine-induced Ischemic Necrosis of the Hand

**DOI:** 10.5811/cpcem.2017.4.33513

**Published:** 2017-07-14

**Authors:** David S. Bostick, Michael T. McCurdy

**Affiliations:** * University of Maryland Medical Center, Department of Emergency Medicine, Baltimore, Maryland; † University of Maryland School of Medicine, Division of Pulmonary and Critical Care, Baltimore, Maryland

## Abstract

This case highlights the rare complication of ischemic hand necrosis following peripheral administration of epinephrine and norepinephrine.

## CASE PRESENTATION

A 60-year-old woman with chronic obstructive pulmonary disease and other co-morbidities presented to the emergency department with dyspnea. Her symptoms were severe enough to require mechanical ventilation. During her stay, she suffered a cardiac arrest during which a total of three separate doses of epinephrine were administered through a peripheral intravenous (IV) catheter on the dorsum of her right hand. Following return of spontaneous circulation she was continued on a norepinephrine infusion through the same peripheral IV catheter for 24 hours for post-arrest hypotension in the setting of septic shock. Two days after her arrest, she was noted to have skin changes consistent with ischemic necrosis of her right hand, which can be seen in the image.

## DISCUSSION

Diagnosis of extravasation injuries can be quite challenging; clinical signs may include pain, swelling, erythema, pain, blistering, or blanching of the skin overlying the site of infusion. Prompt recognition of these signs and appropriate intervention should occur within 4 – 6 hours from the time of injury, although reports have shown benefit of intervening up to 12 hours after injury.^1^ Vigilant monitoring is essential as compartment syndrome can develop after prolonged vasospasm or with large-volume extravasation.^1^ Early management includes simple immobilization, frequent neuromuscular checks, elevation, local application of warming blankets, and injectable or topical vasodilators.^1^,^2^ If unsuccessful, as in this case, open surgical debridement can be used.^2^

Our patient did have local debridement. Despite this, she eventually required amputation at the wrist 43 days after the initial arrest. After a prolonged hospitalization, she was liberated from the ventilator and discharged to subacute rehabilitation.

CPC-EM Capsule

What do we already know about this clinical entity?

Extravasation injuries are challenging to diagnose; however, is important that they are recognized and treated early. This becomes even more important when administering catecholamines peripherally in critically ill patients.

What is the major impact of the image?

It is a reminder that patients receiving catecholamines peripherally are at risk for serious complications.

How might this improve emergency medicine practice?

This image serves as a reminder that it is important to heavily weight the risks and benefits of administering catecholamines through a peripherally inserted intravenous catheter and to discuss the risk of injury with patients or their families.

## Figures and Tables

**Image:**
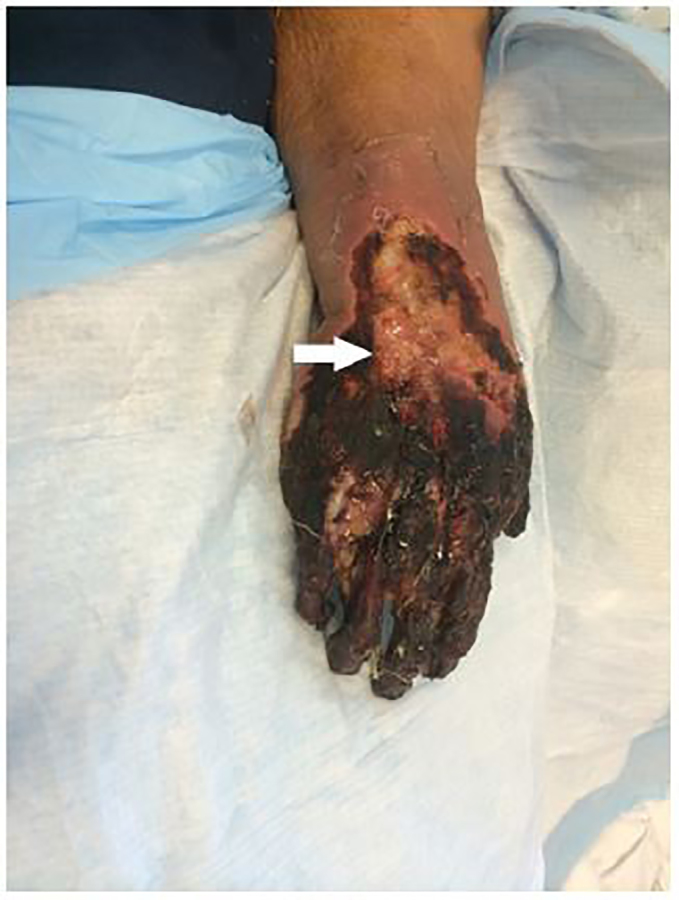
Ischemic necrosis of the right hand following peripheral catecholamine administration.
